# Prediction of Paratope–Epitope Pairs Using Convolutional Neural Networks

**DOI:** 10.3390/ijms25105434

**Published:** 2024-05-16

**Authors:** Dong Li, Fabrizio Pucci, Marianne Rooman

**Affiliations:** 1 Computational Biology and Bioinformatics, Université Libre de Bruxelles, 1050 Brussels, Belgium; dong.li@ulb.be (D.L.); fabrizio.pucci@ulb.be (F.P.); 2 Interuniversity Institute of Bioinformatics in Brussels, 1050 Brussels, Belgium

**Keywords:** antibody design, antibody–antigen complex structures, machine learning

## Abstract

Antibodies play a central role in the adaptive immune response of vertebrates through the specific recognition of exogenous or endogenous antigens. The rational design of antibodies has a wide range of biotechnological and medical applications, such as in disease diagnosis and treatment. However, there are currently no reliable methods for predicting the antibodies that recognize a specific antigen region (or epitope) and, conversely, epitopes that recognize the binding region of a given antibody (or paratope). To fill this gap, we developed ImaPEp, a machine learning-based tool for predicting the binding probability of paratope–epitope pairs, where the epitope and paratope patches were simplified into interacting two-dimensional patches, which were colored according to the values of selected features, and pixelated. The specific recognition of an epitope image by a paratope image was achieved by using a convolutional neural network-based model, which was trained on a set of two-dimensional paratope–epitope images derived from experimental structures of antibody–antigen complexes. Our method achieves good performances in terms of cross-validation with a balanced accuracy of 0.8. Finally, we showcase examples of application of ImaPep, including extensive screening of large libraries to identify paratope candidates that bind to a selected epitope, and rescoring and refining antibody–antigen docking poses.

## 1. Introduction

Antibodies (Ab) are key proteins that play a central role in the immune system. They bind to immunogenic molecules known as antigens (Ag) with high levels of specificity and affinity and trigger different mechanisms of immunity. Their ability to specifically bind antigens (Ag), especially proteins, has made them widely applicable in the diagnosis and treatment of diseases. In particular, the use of monoclonal Abs (mAbs) as therapeutic drugs against cancer and other fatal diseases has increased rapidly in the last two decades and is expected to continue to grow in the coming years [1,2]. Thus, there is an urgent need to develop new and efficient Ab design methods.

An Ab is typically a Y-shaped homodimer of heterodimers, each composed of a heavy (H) and a light (L) chain. The light chain contains a variable and a constant domain (VL and CL), and the heavy chain containing one variable and three heavy domains (VH, CH1, CH2, and CH3). These domains can be divided into two parts. One is the fragment crystallizable (Fc) region that includes CH2 and CH3 domains, which interact with receptors on the surface of phagocytes such as macrophages, neutrophils, and dendritic cells. The other part is the fragment Ag-binding (Fab) region, which is composed of the variable Fv region containing the two variable domains VH and VL, which recognize and bind to antigens, and the constant CL and CH1 domains that structurally support the Fab.

Each Fv region contains six regions with hyper-variable sequences: three in the L-chain and three in the H-chain. They are referred to as complementarity-determining regions (CDRs). As indicated by their names, they contribute to the formation of immune complexes. Despite the sequence variations between the CDRs of different Abs, not all CDR residues participate in Ab-Ag binding, and some residues outside the CDRs were also observed in binding interfaces [3,4,5,6]. Ab residues that are part of the Ab-Ag interface constitute the paratope, and the Ag residues of this interface form the epitope. An Ab can have multiple paratopes, which bind to different epitopes in the same or another Ag [7,8,9].

Several high-throughput screening-based experimental methods for designing Abs against a given Ag have been proposed [10,11,12,13,14,15], but they are time- and resource-consuming. Computer-aided antibody design appears to be a good alternative. Generally speaking, a computational pipeline for Ab design starts by modeling the three-dimensional (3D) structure of complete Abs or Ab fragments, followed by predicting their binding affinity for the target Ag (using, e.g., docking [16,17] and energy functions [18]). Then, selected Abs are optimized in terms of stability, solubility, and binding affinity, either experimentally or computationally [19,20]. The majority of computational methods can be grouped into three categories: (1) designing a complete Ab from scratch [20]; (2) designing parts of an Ab that mainly contribute to its binding with the antigen (usually the paratope or CDR), followed by CDR or paratope grafting onto an Ab scaffold to construct a complete Ab [18,21,22]; and (3) engineering an existing Ab to improve its specificity and affinity or to generate new functions [23,24]. Despite advances in computational Ab design methods, pipelines with a high level of accuracy are not yet available. Besides, tools for paratope design are rare in comparison with complete Ab or CDR design.

We focused on the prediction of paratope–epitope binding. So far, a series of characteristics of paratopes have been identified, such as the over-representation of aromatic residues, especially tyrosine, their tendency to form hydrogen bonds, cation-π, and π-π interactions with the epitope, and a lower propensity to form hydrophobic interactions compared with general protein–protein interfaces [25,26,27]. It was also discovered that paratopes, instead of being rigid interfaces, are characterized by a certain level of flexibility and are able to modify their conformation to some extent during the interaction with Ags [28,29].

In this study, we present ImaPEp, an image-based predictor for paratope–epitope prediction using machine learning (ML) methods and protein structural features. The predictor uses a residual neural network (ResNet) architecture [30] and was trained on a non-redundant dataset of 3D structures of Ab-Ag complexes.

To our knowledge, ImaPEp is the first structure-based computational method for predicting the binding between an Ab and a protein Ag using a two-dimensional (2D) representation of the binding interfaces, i.e., of the paratope–epitope pair. First of all, predicting such pairs rather than single epitope residues and paratope residues is already an improved approach compared to other computational methods [31,32,33,34,35,36]. Moreover, the inclusion of structural information sets ImaPEp aside from sequence-only methods which provide no precise information about the binding residues and the type of interactions [37,38,39], especially as structural motifs at the binding interface are known to be fundamental for Ab-Ag recognition [5]. ImaPEp also differs from other structure-based methods [23,40,41,42], as it considers the entire binding interface with a simplified representation and a shallow neural network as model architecture. This results in a method that is faster and less prone to overfitting than other structure-based approaches, thereby enabling its application to large-scale screening of Ab-Ag binding complexes.

## 2. Results and Discussion

### 2.1. Performance of the Models

**Figure 1:**
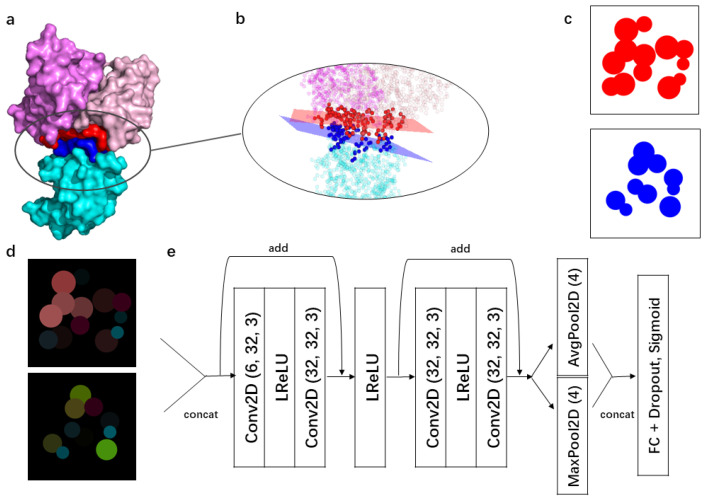
Illustration of the ImaPEp pipeline. (**a**) Example of Ab-Ag complex (PDB ID: 1A2Y), with the Ab light chain colored in light pink, the Ab heavy chain in magenta, and the Ag in cyan; the paratope residues are shown in red and the epitope residues in dark blue. The picture is created using the PyMol package [43]. (**b**) The atoms of paratope residues are shown as red spheres and the epitope atoms as blue spheres. The 3D perspectives were drawn by the vedo package of Python. (**c**) The C_*μ*_ spheres of the paratope and epitope residues projected onto their respective PCA planes are depicted as red and blue circles, respectively. (**d**) The paratope and epitope images are colored according to the physicochemical properties of their residues (RGB color code). (**e**) Each of these two images were converted into 3D tensors, with the first dimension corresponding to the image height (100 pixels), the second to the image width (100 pixels), and the third to the channels (3 RGB colors). These two tensors were then concatenated on the third channel, resulting in a 100 × 100 × 6 tensor. The ensemble of so obtained Ag-Ab tensors from the learning set was used as the training sample. The numbers in parentheses after “Conv2D” represent the number of input channels, the number of output channels, and the convolutional kernel size. The numbers in parentheses after “MaxPool2D” and “AvgPool2D” represent the window size of pooling. “LReLu” represents the leaky-ReLU activation function. “FC + Dropout + sigmoid” refers to a fully connected layer to which the Dropout mechanism is applied and whose output is activated by a sigmoid function. “Add” refers to the addition of two tensors with the same shape.

The ImaPep pipeline for predicting binding paratope–epitope pairs was developed based on the structure dataset $D_{\mathrm{Ab}-\mathrm{Ag}}$ using the CNN model depicted in Figure 1, as described in Section 3. It was tested in 10-fold cross validation where the training was performed on a $D_{\mathrm{subtrain}}$ set and validated on the associated validation set $D_{\mathrm{val}}$. Moreover, it was also tested on an external test set $D_{\mathrm{test}}$. Two versions of the model were developed, ImaPEp-atom and ImaPEp-resi, in which the epitope and paratope images were generated on an atom and residue level, respectively. The metrics used to evaluate the performance of the predictors were the balanced accuracy (BAC) and the Matthews correlation coefficient (MCC), two threshold-dependent metrics, as well as the area under the receiver operating characteristic curve (AUROC) and under the precision–recall curve (AUPRC), two threshold-independent metrics (see Supplementary Section S4 for details). The values of these metrics on $D_{\mathrm{val}}$ and $D_{\mathrm{test}}$ are summarized in Table 1 and detailed in Supplementary Tables S2 and S3.

The first result we want to underline is the good performances of both ImaPEp-resi and ImaPEp-atom. Indeed, both reached a BAC of 0.8, an MCC of 0.6–0.7, an AUROC above 0.9 and an AUPRC of 0.8–0.9 (Table 1). However, ImaPEp-resi performs better than ImaPEp-atom, whatever the metric and test set considered. For example, the BAC and MCC increased from 0.78 to 0.84 and from 0.57 to 0.70, respectively, on $D_{\mathrm{test}}$. This suggests that atomic details are not too informative in this problem and that a coarse-grained model in which the side chain is represented by the C_*μ*_ side chain centroid performs better. Although this is a bit unexpected, as a per-atom representation is supposed to save more details of an Ab-Ag interface, such detailed representation is also likely to introduce noise that could explain the drop in performances.

**Figure 2:**
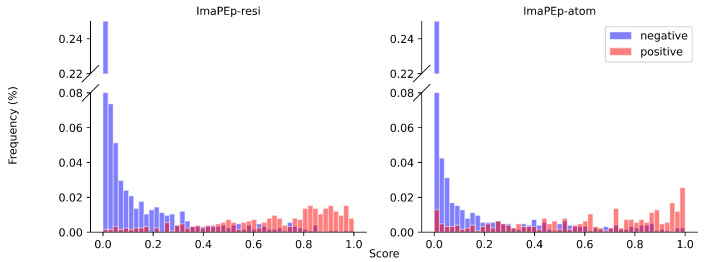
Distributions of scores outputted by ImaPEp-resi and ImaPEp-atom applied to the $D_{\mathrm{test}}$ set.

**Figure 3:**
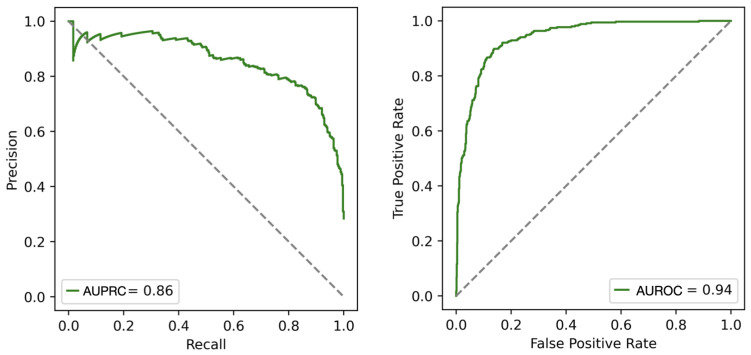
Precision-recall (PR) curve (**left**) and receiver operating characteristic (ROC) curve (**right**) plotted according to the scores outputted by ImaPEp-resi applied to the $D_{\mathrm{test}}$ set; the area under the PR and ROC curves (AUPRC and AUROC) are given.

The good performance of our methods is also demonstrated graphically in Figure 2, where the distribution of both ImaPEp-resi and ImaPEp-atom scores for positive and negative entries in $D_{\mathrm{test}}$ are shown to be clearly separated. The precision–recall (PR) curve (left) and receiver operating characteristic (ROC) curves of ImaPEp-resi are shown in Figure 3.

### 2.2. Ablation Studies

We performed a series of ablation studies in ImaPEp-resi to gain insight into the importance of the different features included in our model and the granularity of the representation. The results are shown in Table 2.

**I. Distance to the PCA plane**. We investigated the impact of considering the contribution of the distance of the residues to the epitope–paratope plane. We trained a model on the images with and without the distance feature. The results in Table 2 show that without the integration of the distance factor, model I performs slightly worse than the standard ImaPEp-resi (BAC 0.81 vs. 0.84). This underlines the importance of geometrical factors in the epitope–paratope recognition.

**II. Coloring system**. To examine whether our model benefits from the physical–chemical information of the residues integrated in the RGB color vector, we generated another kind of color encoding, in which aromatic residues were colored in green, positively charged residues in red, negatively charged residues in blue, and all the other residues in white. We called this the four-color mode. We trained a new model with this new coloring scheme (model II), without changing any hyperparameters and compared its performance with that of the original model. As shown in Table 2, the model trained on the four-color scheme achieved an equal BAC, a slightly higher MCC, but a lower AUROC and AUPRC than the original one, indicating that a finer-grained physical–chemical representation is more informative.

**III. Image size**. Originally, the images used for training were first generated in 200×200 and cropped to 100×100 with respect to the image center. In our dataset, images of this size cover the whole interfaces for the vast majority of the samples. We wondered whether the image size has an effect on the model’s performance. To determine the answer to this, we constructed a new training and test set, which were identical to $D_{\mathrm{train}}$ and $D_{\mathrm{test}}$ except that the images in the new sets have a 64×64 pixel shape. In the dataset, images of this size cover the whole interface for the vast majority of the samples, but the model trained on this dataset has fewer parameters. The results in Table 2 show that the standard ImaPEp-resi model that is trained on images with a larger size performs better than the model trained on reduced images (model III), which indicates that the larger set of parameters in ImaPEp-resi allows for a more accurate learning of Ab-Ag interface peculiarities.

**IV. Biophysical features**. To further investigate the contribution of each individual physical–chemical feature to the model, we ran a series of knock-out experiments, in which we substituted one feature at a time with a tensor of all zeros (knock-out), trained a new model based on the new features, and analyzed the metric values on $D_{\mathrm{test}}$. As shown in Table 2, the original model achieves the best performance even though the differences are not significant. The differences between different knock-out groups of features are also rather small. The model with the isoelectric point knocked out (model IV.b) has the lowest accuracy, suggesting the prominent role of electrostatic interactions in Ab-Ag recognition, which has already been mentioned in the literature [44]. Additionally, when knocking out hydrophobicity (model IV.c), the model works equally well, suggesting, as expected, the minor role of hydrophobic interactions in Ab-Ag complex formation.

### 2.3. ImaPEp Applications

We present here two applications of ImaPEp-resi. In the first, we assessed our tool’s performance for rescoring docking poses, and in the second, for screening paratope libraries to identify those binding to a target epitope.

#### 2.3.1. Rescoring Docking Poses

The docking poses used were obtained from the Dockground unbound docking decoy set 2 [45], which contains poses of over 100 protein complexes. As ImaPEp is specifically designed for Ab-Ag recognition, we only used poses of the 24 available Ab-Ag complexes. For each complex, 100 poses were generated, of which 99 were incorrect and the remaining one was near-native. We compared ImaPEp’s results with a series of scoring functions, including simple energy functions (electrostatics, van der Waals, etc.) and composite docking scoring functions, listed in Table 3. Computation of the different functions is performed by CCharPPI [46]. The score-based ranking of the near-native pose out of the 100 poses, averaged over all 24 Ab-Ag complexes, was computed for ImaPEp-resi and compared with the rankings of the 18 methods assessed. A lower average rank indicates better performance in rescoring docking poses.

As shown in Table 3 and Supplementary Table S4, ImaPEp-resi is able to recognize, for each considered Ab-Ag complex, the near-native docking pose out of the 100 poses with an average rank of 26.7, which outperformed 13 out of the 18 methods tested. ImaPEp-resi successfully predicted the ranking of 6 complexes in the top 5%, and of 10 complexes in the top 20%. Despite not being the best scorer, ImaPEp has the advantage of being extremely fast, as will be discussed in Section 2.5. The reduction in the Ab-Ag interfaces from 3D to 2D sped up and simplified the computations, but led to some information loss.

#### 2.3.2. Paratope Library Screening

The speed of ImaPEp allows broad screening of paratope libraries, in order to identify Ab binders to a given epitope. Here, a paratope library consists of a collection of paratope images generated in the way described in the Methods Section 3.3 using the structures in $D_{\mathrm{Ab}-\mathrm{Ag}}$ dataset. The target epitope was also represented as an image generated in the same way. In addition, the epitope image was rotated 12 times, each time by an angle which is a multiple of 30°. This produced 12 rotated versions of the epitope. Each rotated epitope was combined with each of the paratopes in the library, and each paratope–epitope pair was scored by ImaPEp. A paratope that is predicted to bind with an epitope with an angle 0°, 30°, and −30° (referred to as B_0_, B_+30_ and B_−30_) is regarded as a binder.

For this experiment, we considered MSP1-19 from *Plasmodium falciparum*, a parasite surface protein (PDB ID: 1OB1), which is not found in the training set of ImaPEp. The screening of the entire library of paratope images, containing about 14k candidates, yielded good results. Indeed, our method identified the MSP1-19 paratope binder in position 69 out of the 14k candidates, with, however, a rotation angle of −30°. This means that ImaPEp ranked the real binder in the top 0.5% candidates, which confirmed its ability to prioritize, out of huge paratope libraries, paratopes that potentially bind with the target antigen epitope.

### 2.4. Insights into Prediction Mechanisms

An effective ML-based predictor is expected to learn the molecular mechanisms behind Ab-Ag binding. Deficiency in this ability makes the predictor prone to simply being a “reciter” of the data in the training set, which is a product of overtraining and possesses limited generalization ability. It is thus of paramount importance to inspect whether such a predictor learns true biophysical information.

We generated the feature map learned by our neural network architectures of all samples. During the forward propagation of our model, dot multiplication was performed for each sample between the trained weights of the FC layer and the 1D vector produced by flattening the 25×25×64 tensor (which is the concatenation of two 25×25×32 tensors from the pooling layers), resulting in a scalar logit (or log-odds).

Mathematically, a dot multiplication between two vectors is equivalent to a Hadamard multiplication followed by summing up all elements of the Hadamard product. In order to visualize the feature map, we only retrieved for each input the aforementioned Hadamard product and reshaped it into a 25×25×64 tensor. The tensor is then split into two 25×25×32 tensors, corresponding to the maximum and average pooling layers, respectively. A maximum unpooling and average unpooling operation is applied to each of the 25×25×32 tensors, resulting in two 100×100×32 tensors, of which the mean along the third dimension is calculated, generating a 100×100 tensor, which is the ultimate feature map for all convolutional layers.

**Figure 4:**
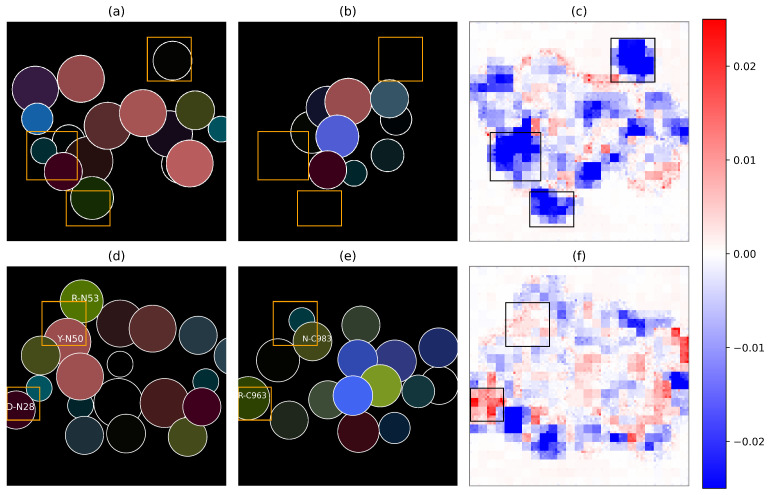
Paratope and epitope images and convolutional feature maps. (**a**) Paratope image of PDB ID 4I77; (**b**) epitope image of PDB ID 1QGC; (**c**) backward feature map of negative sample 4I77-1QGC; (**d**) paratope image of 1FE8; (**e**) epitope image of PDB ID 1FE8; (**f**) backward feature map of the positive sample 1FE8. In the feature maps, red dots represent positive contributions, and blue dots represent negative contributions to the final output.

We showed that ImaPEp-resi is able to extract the contours of the paratope and epitope in a pair, and that the shape compatibility is utilized as an important feature to distinguish between bound and unbound paratope–epitope pairs. A typical example is shown in Figure 4a–c, where the paratope comes from an Ab against cytokine interleukin 13 (IL-13) (PDB ID: 4I77) [47] and the epitope comes from a mouth disease viral protein (PDB ID: 1QGC) [48]; this pair represents a negative data sample. We clearly see in the Figure that the regions where one of the two interfaces has residues and the other does not make negative contributions to the overall binding score, regardless of whether the existing residues are close to or distant from their binding partner.

ImaPEp-resi is also able to recognize information about the non-covalent interactions between residues that contribute to stabilizing the Ab-Ag interface. This is shown in Figure 4d–f, in which the paratope and epitope are obtained from a complex of low-density lipoprotein receptor-related protein 6 (LRP6) and its cognate antibody [49] (PDB ID: 1FE8; Ab-heavy and -light chains are referred to as J and N, and the Ag chain is referred to as C). Indeed, the region outlined in the top box of the images contains an amino-π interaction occurring between Tyr (N50) in the Ab and Asn (C983) in the Ag, and a side-chain–side-chain hydrogen bond between Asn (C983) and Arg (N53). In the bottom box, we have a salt bridge between Asp (N28) and Arg (C963). These two interactions, especially the salt bridge, stabilize the Ab-Ag interface and appear in the feature map as a red area, signaling their important role which is learned by the ImaPEp model.

To gain insights into the relatively poorer performance of the per-atom model compared to the per-residue model, we looked for the entries to which ImaPEp-atom assigned incorrect scores and examined their feature maps. As discussed in Supplementary Section S6 and Figure S1, we found that the per-atom model has an oversensitivity to the distance feature and that its fine-granularity increases the probability of shape incompatibility of the 2D represented interfaces. These behaviors are likely to lead to a loss in performances compared to the residue-based model, which is fully coarse-grained both at the level of the residues and interface representations.

### 2.5. Computational Efficiency

By reducing the 3D Ab-Ag interface to a 2D representation, our model is exempt from the time-consuming 3D graphical computations and thus is computationally efficient. Training 10 cross-validated models takes ~55 graphic processing unit (GPU) minutes (with GPU Tesla P100). The predictions are, moreover, very fast. The scoring of 1 k testing samples (images) costs ~15 GPU seconds and the rescoring of 2.4 k docking poses (including the process of image generation) costs ~1 GPU minute.

## 3. Materials and Methods

### 3.1. Dataset of Ab-Ag Complexes

The dataset that we used in this study was obtained by downloading structures of Ab-Ag complexes from the SAbDab structural antibody database [50], in which the Ab structures deposited in the Protein Data Bank (PDB) [51] have their chains renumbered in a consistent way. We split the PDB files containing multiple Ab-Ag complexes into smaller PDB files, each containing a single Ab-Ag complex. We then filtered this dataset based on the following principles:

- Only true Ab-Ag complexes were retained; we thus removed complexes where the Ab bound to proteins serving as crystallization facilitators.
- Only the complexes containing the whole Fab region or their variable Fv part were kept; Ab derivatives such as single-chain Fvs, nanobodies, etc., were not considered.
- Only complexes in which the Ag was a protein and which contained ≥50 residues were retained.
- All complexes whose structures were solved by X-ray crystallography or electron microscopy were retained, without any threshold for their resolution.
- Complexes that have more than 50 residues with missing atomic coordinates were removed; for the remaining complexes, PDBFixer [52], a Python package for processing PDB files, was used to add the missing atoms.
- Complexes with unknown residues (with identifier “UNK” in the PDB file) were discarded.
- Only complexes with an Ab-Ag binding region (as defined in the next section) that contains at least three residues were selected.

We further refined the set of Ab-Ag complexes obtained after applying these filters on the basis of sequence identity. To this end, we began by identifying the CDRs in the Abs, based on a structure-based definition [53,54,55], which includes all Ab residues in the loops at the Ag interface. This was performed using abnumber, a Python package wrapping ANARCI, which is a tool for CDR identification [56]. For the sequence identity filter, we used CD-HIT [57,58], a computational tool for protein sequence clustering. We separately clustered the sequences of the Ab CDRs and of the Ags. The sequence identity cut-offs were 0.8 for CDR sequences and 0.9 for antigen sequences, which means that two CDR sequences with sequence identity over 0.8 or two Ag sequences with sequence identity over 0.9 were clustered together and considered to be similar (see Supplementary Section S1 for details). To identify similar Ab-Ag complexes, we applied the following procedure: given two Ab-Ag complexes $\left({\mathrm{Ab}}_{1},{\mathrm{Ag}}_{1}\right)$ and $\left({\mathrm{Ab}}_{2},{\mathrm{Ag}}_{2}\right)$, if ${\mathrm{Ab}}_{1}$ is similar to ${\mathrm{Ab}}_{2}$ and ${\mathrm{Ag}}_{1}$ is similar to ${\mathrm{Ag}}_{2}$, the two complexes were considered similar and placed in the same group. In each group, the complex with the longest Ag sequence was retained and all the others were discarded.

In this way, we collected a dataset of 1767 complexes, referred to as $D_{\mathrm{Ab}-\mathrm{Ag}}$. Their PDB structures are available in our GitHub repository https://github.com/3BioCompBio/ImaPEp (accessed on 1 April 2024).

### 3.2. Identification of Paratope and Epitope Residues

We used a distance criterion to identify the Ab-Ag interface. Specifically, a residue in the Ab (Ag) was regarded as paratope (epitope) if it had a distance of ≤6 Å from any residue in the Ag (Ab). We defined the distance between two residues as the distance between their C_*μ*_ pseudoatoms, where the coordinates of a residue’s C_*μ*_ is the mean of all its heavy side-chain atoms. For Gly, the C_*μ*_ coincides with the C_*α*_.

Some Ags are huge proteins which contact Ab residues not only in the conventional Ab-Ag binding region around the CDRs, but also in some other surface regions. Residues in these regions should not be regarded as paratopes or epitopes. To prevent their inclusion, residues with a distance >40 Å from the geometrical center of the CDRs were removed from the list of epitope and paratope residues.

### 3.3. Image Representation of Paratopes and Epitopes

For each Ag-Ab complex, we extracted the 3D coordinates of all the heavy atoms of the paratope and epitope residues, as well as their C_*μ*_ pseudoatom [59], which was defined as the average geometric center of all their heavy side-chain atoms. We then performed principal component analysis (PCA) separately on the 3D coordinates of the paratope and epitope to reduce them to 2D patches. We thus obtained a 2D image of the paratope and a 2D image of the epitope of each Ag-Ab complex. This is illustrated in Figure 1a–c. The details are given in Supplementary Section S2 and Table S1.

We constructed two type of models: the first is coarse-grained with 2D images obtained considering only the C_*μ*_ pseudoatoms of each residue; the radii of the circles centered on the C_*μ*_ pseudoatoms represent the residue sizes [60]. We call this model ImaPEp-resi. The second model, ImaPEp-atom, is defined at the atomic level and considers all heavy side-chain atoms, with each atom represented as a circle of 1 Å radius.

In a second step, an RGB-based bitmap image was generated for each of these so-defined paratope and epitope 2D images. The circles were colored according to the physicochemical properties of the residues; in the atomic model, all atoms of a residue are of the same color. Inspired by the fact that hydrophobicity is a key biophysical property for protein stability and binding, and that aromatic and charged interactions are crucial at Ag-Ab interfaces [25], we chose the following features:

- polarizability (P), as defined in [61].
- isoelectric point (I), as defined in [62];
- hydrophobicity (H), as defined in [63].
- distance (D) to the PCA plane, as defined in Supplementary Section S2.

The first three features correspond to the three channels in the RGB coloring system. The images were generated using the matplotlib package [64]. The distance feature is represented by the transparency of the circles in the images. An example of epitope and paratope images colored according to this scheme is illustrated in Figure 1d. Note that we tested other types of residue information and different encoding schemes, but the simple features we selected emerged as the most relevant because they are intrinsically linked to the biophysics of Ab-Ag binding.

### 3.4. Construction of the Learning, Validation and Test Sets

All complexes in our $D_{\mathrm{Ab}-\mathrm{Ag}}$ dataset were randomly shuffled and split into a training set ($D_{\mathrm{train}}$) containing 80% of the complexes and a test set ($D_{\mathrm{test}}$) containing the remaining 20% of the complexes. Moreover, the training set $D_{\mathrm{train}}$ was randomly split into a subtraining set ($D_{\mathrm{subtrain}}$) containing 90% of the samples and a validation set ($D_{\mathrm{val}}$) containing the remaining 10%. The separation was performed in a stratified way to ensure that both $D_{\mathrm{subtrain}}$ and $D_{\mathrm{val}}$ contain the same proportion of positive and negative samples. The model was trained on $D_{\mathrm{subtrain}}$ and validated on $D_{\mathrm{val}}$ in a 10-fold cross-validation procedure, and finally tested on $D_{\mathrm{test}}$.

In both $D_{\mathrm{train}}$ and $D_{\mathrm{test}}$, cognate Ab-Ag complexes were regarded as positive samples. The negative samples were generated by three mechanisms:

- Pairing non-cognate Abs and Ags;
- Rotating the images of cognate Ab-Ag pairs by different relative angles;
- Translating the images of cognate Ab-Ag pairs by different relative displacements.

The negative samples were obtained using Abs and Ags that are in the same set, which basically guarantees the isolation of the training and test sets. More details are given in Supplementary Section S3.

Note that the $D_{\mathrm{Ab}-\mathrm{Ag}}$ set is unbalanced, as it contains far more negative samples than positive ones. However, this imbalance does not have a significant impact on the model’s performance. Indeed, we trained ImaPEp on various datasets in which the negative entries were downsampled, and the resulting performances showed no statistically significant difference compared to those of the model trained on $D_{\mathrm{Ab}-\mathrm{Ag}}$.

### 3.5. Model Architecture

We used a convolutional neural network (CNN) as model architecture. Specifically, we considered residual network (ResNet) blocks [65], which include a skip-connection mechanism that has proven efficient to stabilize gradient-based model training and resolve the problem of model degradation that is frequently encountered in the training of super deep networks. The precise model structure used for ImaPEp is illustrated in Figure 1e.

The first component of our model is two stacked ResNet blocks [65] connected by a leaky rectified linear unit (LReLU) activation function [66]:

(1) $$\mathrm{LReLU}\left(x\right)=\left\{\begin{array}{cc} x & x>0\\ 0.1x & x\leq 0 \end{array}\right.$$

Each ResNet block contains two convolutional layers connected by LReLU activation. For each sample, the two images were loaded into 100×100×6 tensors and concatenated along the dimension representing the color, resulting in a six-channel “image”, which is represented as follows:

(2) $$X=\left(\begin{array}{ccc} x_{1,1} & ... & x_{1,n}\\ ... & ... & ...\\ x_{n,1} & ... & x_{n,n} \end{array}\right)$$

where $x_{i,j}$ represents the “pixel” at the *i*-th row and the *j*-th column, which is a six-element 1D vector. The convolutional operation used in our model is formulated as follows:

(3) $$c_{i,j}=\sum_{t=-k}^{k}\sum_{s=-k}^{k}F_{t,s}\cdot x_{i+t,j+s}$$

where *F* is the convolutional kernel with a size of 2k+1; here, we used k=1. $c_{i,j}$ is the result of convolution at position (i,j) of the tensor output by the convolutional layer and is called the feature map. During each convolutional operation, the input tensor is padded to make sure the shape is consistent before and after the operation. For each convolutional layer, we used 32 kernels. Each kernel generates one feature map of the same size as the others, which is further activated by a LReLU function.

Immediately after each ResNet block, two pooling layers (one max-pooling and one average-pooling) were introduced to perform the downsampling of the output feature map, which reduced the amount of the trainable parameters and prevented overfitting. The max-pooling operation is formulated as follows:

(4) $$\begin{array}{cc} p_{i,j} & =\max\left(\{r_{(i-1)s+t,(j-1)s+t}|t=1,2,\ldots ,s\}\right) \end{array}$$

and the average pooling operation is formulated as follows:

(5) $$\begin{array}{cc} p_{i,j} & =\mathrm{avg}\left(\{r_{(i-1)s+t,(j-1)s+t}|t=1,2,\ldots ,s\}\right) \end{array}$$

where *s* is the window size of the pooling layer (here, we chose s=4), *r* represents the input to the pooling layer, and $p_{i,j}$ is the pooling result at position (i,j). The result tensors of the two pooling operations were concatenated by the first dimension, which was then flattened to a 1D tensor and fed into a fully connected (FC) layer. The output was mapped by a sigmoid function into scores in the [0,1] range

(6) $$\begin{array}{cc} \sigma (x) & =\frac{1}{1+e^{-x}} \end{array}$$

(7) $$\begin{array}{cc} \hat{y} & =\sigma (w\cdot f+b) \end{array}$$

where *w* is the weight matrix of the FC layer, *f* is the flattened 1D tensor, and *b* is the bias term; $\hat{y}$ is the final output of the network, i.e., the score of the binding of the input paratope–epitope pair. We also added a regularization mechanism to the FC layer, i.e., Dropout [67], which cancels the contribution of some neurons towards the next layer in order to adjust the model’s capacity and achieve a balanced point between overtraining and undertraining.

To obtain an optimal architecture, we manually pruned hyperparameters on $D_{\mathrm{train}}$. We varied the number of ResNet blocks, the number of kernels in each convolutional layer, the position of max-pooling layers, and the dropout rate. Our final model uses two ResNet blocks, with each convolutional layer containing 32 kernels. The dropout rate was set at 0.75.

### 3.6. Training

To validate the performance of our model, 10-fold stratified cross-validation was performed on $D_{\mathrm{train}}$, with $D_{\mathrm{subtrain}}$ and $D_{\mathrm{val}}$ serving as training and validation sets, respectively, as described in Section 3.4. We introduced an early stopping mechanism to the training [68], which observes the performance metrics on $D_{\mathrm{val}}$ and terminates the training if their values plateau or worsen on $D_{\mathrm{val}}$ for a predefined period. We used binary cross-entropy as the loss function:

(8) $$J\left(\theta \right)=-\frac{1}{m}\sum_{i=l}^{m}\left[y_{l}\ln\hat{y_{l}}+\left(1-y_{l}\right)\ln\left(1-{\hat{y}}_{l}\right)\right]$$

where ${\hat{y}}_{l}$ is the model’s score of the $l^{\mathrm{th}}$ sample (with the label $y_{l}$) outputted by the model with parameters θ; *m* is the number of samples. Adam [69] was adopted as the optimizer. A learning rate scheduler was introduced along with Adam. Similar to early stopping, it also works by observing the performance on $D_{\mathrm{val}}$, but adjusts the learning rate instead of terminating training in case the performance does not progress. For the initialization of the model parameters, we used the Kaiming normal initialization method [65] for all the convolutional layers, as it is a specific initialization method designed for layers with LReLU activation. We initialized the weights of the FC layer by Xavier normal initialization [70] as this method initializes the weights such that the variance of the activations are the same across every layer, which has proven beneficial for the convergence of the training. During each training epoch, a mini-batch of samples was sequentially fed to the model.

After the 10 models of the cross-validation procedure were generated, the ultimate score was calculated as the mean value of the scores outputted by the models. All the programs in ImaPEp were developed in Python using the PyTorch framework.

## 4. Conclusions

In this study, we introduced a 2D image-based predictor called ImaPEp, which is capable of predicting the binding probability of a given paratope and epitope pair with considerable performance. We analyzed the features contributing to the per-residue representation model, which achieved slightly higher accuracy compared to the per-atom model. Despite their simplicity, the biophysical properties of the interacting residues provide a physiochemistry-based coloring scheme with good model performance. The distance of each interface residue to its binding partner also plays an indispensable role in predicting the Ab-Ag binding, in addition to residue biophysical properties. Finally, we demonstrated that the prediction of ImaPEp significantly depends on the complementarity between the shapes of the paratope and epitope.

However, the ImaPEp model also has limitations. From the dataset perspective, one of the well-known difficulties is the generation of negative learning samples. As no incorrectly bound Ab-Ag structures can be experimentally determined, we have to rely on artificial negative samples. The choice of this negative set can restrict the learning of relevant features from the positive set of Ab-Ag complexes. Moreover, the vast number of non-cognate epitope–paratope pairs used in our negative sample can render the dataset extremely unbalanced. Additionally, the low specificity of some Abs, which implies that some paratopes can bind to more than one epitope, albeit with a different affinity, also introduces noise into the positive training dataset.

Another limitation of ImaPEp lies in the reduction of the 3D structure of Ab-Ag interfaces to 2D images. This inevitably leads to some loss of information and inaccuracies in the distance representation, as shown in Supplementary Section S7, Figure S2 and Table S5.

Despite these limitations, our method achieves very good performance, as demonstrated in both the binary binding epitope–paratope pair classification and the additional tests we conducted, such as the rescoring of the docking poses. The loss of information in our representation has the advantage of allowing our method to be very fast, as demonstrated in the computational screening of tens of thousands of paratopes to identify the correct binding partner for the selected epitopes.

Future development will involve creating negative sets of paratope–epitope pairs which do not bind but are sufficiently challenging to distinguish from positive samples for ML models. This will certainly lead to an increase in the robustness of the models and their performance. Moreover, other representations of Ab-Ag interfaces, involving intricate geometrical features, are also conceivable and are expected to improve the results achieved with our current model based on a simplified 2D representation. Finally, combining ImaPEp with slower scoring approaches that use less simplified, atomistic representations (see, e.g., [71]) could also lead to an improved prediction pipeline for antibody design.

In summary, we are confident that our ImaPEp computational method will be of interest to the entire scientific research community working on optimizing Ab-Ag complexes. It could accelerate the discovery of new antibody-related drugs, addressing one of the most intriguing and pressing challenges in the biomedical field today.

## Figures and Tables

**Table 1:** Balanced accuracy (BAC), Matthews correlation coefficient (MCC), area under the receiver operating characteristic curve (AUROC) and under the precision–recall curve (AUPRC) of the per-residue and per-atom pipelines ImaPEp-resi and ImaPEp-atom applied to the external test set $D_{\mathrm{test}}$.

Model	BAC	MCC	AUROC	AUPRC
ImaPEp-resi	0.84	0.70	0.94	0.86
ImaPEp-atom	0.78	0.57	0.90	0.77

**Table 2:** Ablation studies in ImaPEp-resi. We trained six models: (I) without the distance-based color reduction mechanism. ✓ and X respectively indicates the introduction and removal of the distance feature; (II) with different image coloring scheme; (III) with images of different size; (IV.a) with polarizability (P) knocked out; (IV.b) with isolelectric point (I) knocked out; (IV.c) with hydrophobicity (H) knocked out.

Model	Shape	Distance	Features	BAC	MCC	AUROC	AUPRC
ImaPEp-resi	100×100	✓	P-I-H	0.841	0.697	0.940	0.857
I	100×100	X	P-I-H	0.813	0.651	0.927	0.830
II	100×100	✓	4-color	0.841	0.699	0.935	0.835
III	64×64	✓	P-I-H	0.799	0.614	0.905	0.775
IV.a	100×100	✓	X-I-H	0.836	0.683	0.936	0.839
IV.b	100×100	✓	P-X-H	0.822	0.678	0.933	0.839
IV.c	100×100	✓	P-I-X	0.850	0.717	0.943	0.855

**Table 3:** Ranking of the score of the near-native docking pose out of all 100 poses, averaged over 24 Ab-Ag complexes, using ImaPEp-resi and 18 other methods [46]. A lower rank indicates a better prediction.

Scoring Function	Average Rank
ImaPEp	26.7
ELE	24.5
HBOND2	42.9
VDW	62.2
AP_DCOMPLEX	43.2
AP_DFIRE2	25.7
AP_PISA	29.2
AP_dFIRE	29.5
AP_T2	49.5
CP_PIE	80.1
LK_SOLV	60.9
FIREDOCK	22.6
FIREDOCK_AB	57.5
FIREDOCK_EI	22.8
ROSETTADOCK	61.5
ZRANK	40.9
ZRANK2	58.5
PYDOCK_TOT	17.3
SIPPER	94.1

## Data Availability

The programs and data are available in our GitHub directory https://github.com/3BioCompBio/ImaPEp (accessed on 1 April 2024).

## Supplementary Materials

The following supporting information can be downloaded at https://www.mdpi.com/article/10.3390/ijms25105434/s1. References [45,46,57,58,59,60,61,62,63,72] are cited in the Supplementary Materials.
