# Supplementary material for: Prediction of Paratope–Epitope Pairs Using Convolutional Neural Networks
Source: Int J Mol Sci. 2024 May 16;25(10):5434. doi: 10.3390/ijms25105434 (PMC11121317; doi:10.3390/ijms25105434)
Supplement: Supplementary file 1 [file ijms-25-05434-s001.zip › ijms-2970998-supplementary.pdf]

Supplementary Material

Prediction of Paratope-Epitope Pairs Using Convolutional Neural Networks

Dong Li, Fabrizio Pucci, Marianne Rooman
Computational Biology and Bioinformatics, Université Libre de Bruxelles, Brussels, Belgium

Table of content

- Section S1. Clustering antibody and antigen sequencess
- Section S2. Image generation
- Section S3. Dataset construction
- Section S4. Evaluation metrics
- Section S5. Tables and figures
- Section S6. Comparison of per-residue and per-atom models
- Section S7. 3D distances in the 2D representation

S1 Clustering antibody and antigen sequences

We used CD-HIT [1, 2] to cluster the sequences, with the cutoff set to 0.8 for the concatenated sequences of antibody CDRs, and 0.9 for antigen sequences. We used the command-line interface (CLI) of CD-HIT. The commands we used for sequence clustering are:

```
# command for antibody
cd-hit -i ab.fasta -o ab -c 0.8 -T 0 -d 0 -l 4 -sc 0 -G 0 -g 1 -p 1 -aS 0.9
# command for antigen
cd-hit -i ag.fasta -o ag -c 0.9 -T 0 -d 0 -l 4 -sc 0 -G 0 -g 1 -p 1 -aS 0.9
```

Note that CD-HIT can give different results even with exactly the same input parameters.

S2 Image generation

For each patch, the four boundaries, i.e. the maximum and minimum values of the atom or residue coordinates along the x-axis and y-axis were determined. Based on these, we defined a frame able to fully enclose the patch. The left (top) boundary of the frame was computed as the largest number which is a multiple of 5 and smaller than the left (top) boundary of the patch. Similarly, the frame's right (bottom) boundary was computed as the smallest number which is a multiple of 5 and larger than the right (bottom) boundary of the patch. For most patches, the frame's boundaries are -25Å on the top and left and +25Å on the right and bottom, i.e. the frame is a 50Å × 50Å square. For the patches with a different frame, padding or cropping was applied.

If the radius of an atom is set to 1Å and if we take a 1:1 Å-pixel ratio in ImaPEp-atom's image, an atom will likely take up less than a pixel of space. We thus decided to utilize a 1:4 Å-pixel ratio. As a result, all patch images have 200 × 200 pixels. The resulting image contains many blank regions around the colored area. To reduce the model complexity we cropped all the images to 100 × 100 pixels.

In ImaPEp-atom, we used the same size for the circles representing the atoms. The van der Waals radii of oxygen, nitrogen, carbon and sulfur atoms are equal to 0.60, 0.65, 0.70

Table S1: Radius (in Å) [3], polarizablity [4], isoelectric point [5], and hydrophobicity (Kyte-Doolittle scale [6]) of the 20 amino acid residues. The min-max scaled values are given in parentheses.

Residue	Radius	Polarizability	Charge	Hydrophobicity
Gly	1.9	0.03 (0.0)	6.06 (0.4)	-0.4 (0.46)
Ser	2.4	1.6 (0.13)	5.7 (0.35)	-0.8 (0.41)
Thr	2.8	2.7 (0.22)	5.6 (0.34)	-0.7 (0.42)
Ala	2.3	1.1 (0.09)	6.11 (0.40)	1.8 (0.7)
Val	2.91	3.2 (0.26)	6.02 (0.39)	4.2 (0.97)
Leu	3.15	4.2 (0.35)	6.04 (0.39)	3.8 (0.92)
Ile	3.09	4.3 (0.35)	6.04 (0.39)	4.5 (1.0)
Cys	2.5	2.7 (0.22)	5.15 (0.28)	2.5 (0.78)
Met	3.1	5.1 (0.42)	5.71 (0.35)	1.9 (0.71)
Phe	3.4	8.0 (0.66)	5.76 (0.36)	2.8 (0.81)
Asp	2.8	3.0 (0.25)	2.98 (0.0)	-3.5 (0.11)
Glu	3.05	4.1 (0.34)	3.08 (0.01)	-3.5 (0.11)
Asn	2.85	3.7 (0.3)	5.43 (0.31)	-3.5 (0.11)
Gln	3.05	4.8 (0.4)	5.65 (0.34)	-3.5 (0.11)
Pro	2.8	4.3 (0.35)	6.3 (0.43)	-1.6 (0.32)
His	3.1	6.3 (0.52)	7.64 (0.6)	-3.2 (0.14)
Lys	3.15	5.2 (0.43)	9.47 (0.83)	-3.9 (0.07)
Arg	3.16	8.5 (0.7)	10.76 (1)	-4.5 (0)
Tyr	3.45	8.8 (0.73)	5.63 (0.34)	-1.3 (0.36)
Trp	3.6	12.1 (1)	5.88 (0.37)	-0.9 (0.4)

and 1.00 Å, respectively [7], and thus cover 3 or 4 pixels. For simplicity, we considered all atom radii equal to 1 Å or 4 pixels. All atoms of the same residue were colored in the same way, according to their polarizability, isoelectric point and hydrophobicity. The values of polarizability and isoelectric points were directly taken from [4] and [5], respectively. For hydrophobicity, we used the Kyte-Doolittle scale [6]. Each of the three metrics were min-max scaled to the [0-1] range; see Table S1 for more details.

In ImaPEp-resi, we represented each residue as a solid circle centered on the C_μ pseudoatom [8] defined as the average of the coordinates of all its heavy side chain atoms. The radius of the circles are related to the residue size; we took the values of [3]. The coloring scheme is the same as the one used in ImaPEp-atom.

Moreover, we introduced a distance-based color reduction mechanism. We computed a "signed distance" between the 3D coordinates of each atom/residue and the paratope-epitope PCA plane. Normally, the PCA plane lies between the paratope and epitope and we artificially defined its side in the direction of most epitope atoms/residues as the "epitope side" and the other as the "paratope side". Paratope atoms/residues located at the epitope side were considered as closer to the epitope and their distances were considered as positive, whereas the sign of those at the paratope side were negative; and vice versa for the epitope atoms/residues. The signed distances were mapped by sigmoid functions to squeeze them to the [0-1] range, generating a series of [0-1] scaled distance-based values, which were used as color reduction coefficient multiplying the original RGB vectors to finalize the colors used in 2D pictures. With this color-reduction system, atoms/residues which are more distant from the binding partner are represented in lighter colors. Moreover, if two atom/residues have an overlap in the image, the one with a more positive distance is put on top of the other.

S3 Dataset construction

Negative samples were created separately for the training set and the test set. These samples were generated by three mechanisms:

- **Non-cognate antibody-antigen pairing.** We first took the non-redundant antigen set (in which antigens have a pairwise sequence identity of 90% at most), and chose for each antigen up to three non-cognate antibodies from the non-redundant antibody set (in which CDR sequences have a pairwise sequence identity of 80% at most) to pair with it. To increase the level of difficulty, we further clustered the non-redundant antibody sequences with a cutoff of 60%. For each antigen, all the non-cognate antibodies we chose have a sequence identity over 60% and below 80%.

- **Interface rotation**. For each paratope-epitope image pair, one image was rotated with respect to the other to create a negative sample. To further increase the level of difficulty of the learning task, we chose a relatively small rotational angle, which was randomly and uniformly sampled from 20° to 40°. Considering that a typical antibody-antigen interface has an area of about 1000 $Å^2$ [7], and assuming the interface is a circle, we can estimate it has a radius of about 15 Å. Thus, when an interface is rotated by 20° to 40°, each point on the edge of the circle is displaced by 5-10 Å, as calculated with the formula:
$$L = 2 \times a \times \sin(\theta/2) \quad \text{(S1)}$$
where a denotes the length of the axis of rotation and θ is the rotational angle. This range of displacements appears to be large enough to destroy the interactions originally formed in the antibody-antigen interface.

- **Interface translation**. In each image pair, one image was translated with respect to the other. With the same objective as for interface rotations, the displacements were constrained to relatively small values. We translated one of the images of a pair both along the x-axis and y-axis by a displacement uniformly sampled from 8 to 16 pixels. As mentioned above, 4 pixels in our images are equivalent to 1 Å. So, a displacement of 8 to 16 pixels along one axis is equivalent to a translation of 2 to 4 Å. For simplicity, we regarded the equivalent displacement in the 3D space as $2\sqrt{3}(\approx 3.5)$ Å to $4\sqrt{3}(\approx 6.9)$ Å, which is sufficient to destroy the interactions.

S4 Evaluation metrics

The most commonly used threshold-dependent and threshold-independent metrics for binary classifications were used in this study: balanced accuracy (BAC), Matthews's correlation (MCC), area under receiver operating characteristic curve (AUROC) and area under precision-recall curve (AUPRC). These metrics are defined as follows, where TP, TN, FP and FN are true positives, true negatives, false positives and false negatives, respectively:

- BAC= $\frac{1}{\text{TPR+TNR}}$, where TPR= $\frac{\text{TP}}{\text{TP+FN}}$ and TNR= $\frac{\text{TN}}{\text{TN+FP}}$. BAC values are between 0 and 1, with 0.5 being the random value and 1 the perfect value.

- MCC= $\frac{\text{TP}\times\text{TN}-\text{FP}\times\text{FN}}{\sqrt{(\text{TP+FP})(\text{TP+FN})(\text{TN+FP})(\text{TN+FN})}}$. MCC values are beween -1 and 1, with 0 being the random value and 1 the perfect value.

- AUROC: The receiver operating characteristic curve (ROC) curve is the plot of the true positive rate (TPR) as a function of the false positive rate (FPR= $\frac{\text{FP}}{\text{FP+TN}}$) at each threshold setting. All predicted scores are considered as thresholds values. The AUROC value is the area under ROC curve. It is between 0 and 1, with 0.5 being the random value and 1 the perfect value.

- AUPRC: The precision-recall curve (PRC) is the plot of the precision or positive predictive value (PPV= $\frac{\text{TP}}{\text{TP+FP}}$) as a function of the recall or TPR at each threshold setting. The AUPRC value is the area under PRC curve. It is between 0 and 1, with 0.5 being the random value and 1 the perfect value.

S5 Tables and Figures

Table S2: Balanced accuracy (BAC), Matthews correlation coefficient (MCC), area under the receiver operating characteristic curve (AUROC) and under the precision-recall curve (AUPRC), computed in 10-fold cross validation, using the per-atom pipeline ImaPEp-atom. Each single model was trained on a $\mathcal{D}_{\text{subtrain}}$ set and applied to the corresponding $\mathcal{D}_{\text{val}}$ set and the independent $\mathcal{D}_{\text{test}}$ set.

	$\mathcal{D}_{\text{val}}$		$\mathcal{D}_{\text{test}}$			
Model	BAC	MCC	BAC	MCC	AUROC	AUPRC
1	0.68	0.40	0.80	0.62	0.90	0.77
2	0.65	0.37	0.75	0.54	0.89	0.74
3	0.69	0.40	0.79	0.58	0.89	0.75
4	0.60	0.22	0.75	0.54	0.89	0.74
5	0.72	0.45	0.76	0.53	0.89	0.74
6	0.65	0.32	0.75	0.51	0.88	0.72
7	0.70	0.45	0.75	0.54	0.88	0.73
8	0.66	0.36	0.76	0.53	0.88	0.73
9	0.70	0.43	0.76	0.54	0.89	0.75
10	0.67	0.36	0.77	0.56	0.89	0.74

Table S3: Balanced accuracy (BAC), Matthews correlation coefficient (MCC), area under the receiver operating characteristic curve (AUROC) and under the precision-recall curve (AUPRC), computed in 10-fold cross validation, using the per-residue pipeline ImaPEp-resi. Each single model was trained on a $\mathcal{D}_{\text{subtrain}}$ set and applied to the corresponding $\mathcal{D}_{\text{val}}$ set and the independent $\mathcal{D}_{\text{test}}$ set.

	$\mathcal{D}_{\text{val}}$		$\mathcal{D}_{\text{test}}$			
Model	BAC	MCC	BAC	MCC	AUROC	AUPRC
1	0.77	0.58	0.82	0.64	0.92	0.81
2	0.75	0.50	0.83	0.65	0.92	0.82
3	0.73	0.48	0.81	0.65	0.93	0.83
4	0.81	0.61	0.83	0.64	0.91	0.79
5	0.68	0.43	0.75	0.58	0.92	0.81
6	0.72	0.49	0.76	0.60	0.92	0.82
7	0.74	0.54	0.81	0.65	0.92	0.83
8	0.78	0.54	0.83	0.64	0.92	0.80
9	0.82	0.65	0.83	0.66	0.93	0.82
10	0.81	0.62	0.84	0.66	0.92	0.80

Table S4: Performance of ImaPEp-resi and 18 selected scoring functions (computed using CCharPPI [9]) on the docking poses of the Dockground dataset [10]. Each value in the TOP 5% column represents the number of antibody-antigen complexes (out of 24) for which the model ranks the near-native pose within the top 5%. Similarly, the TOP 10% and TOP 20% columns indicate the number of complexes for which the near-native pose is ranked within the top 10% and 20%, respectively. The average rank column displays the average ranking of the near-native pose across the 24 complexes considered.

Scoring Function	TOP 5%	TOP 10%	TOP 20%	Average Rank
ImaPEp	6	6	10	26.7
ELE	8	11	13	24.5
HBOND2	2	3	8	42.9
VDW	3	4	4	62.2
AP_DCOMPLEX	3	4	7	43.2
AP_DFIRE2	7	11	14	25.7
AP_PISA	7	10	14	29.2
AP_dFIRE	8	11	13	29.5
AP_T2	1	1	4	49.5
CP_PIE	0	1	1	80.1
LK_SOLV	0	0	0	60.9
FIREDOCK	4	8	16	22.6
FIREDOCK_AB	0	1	1	57.5
FIREDOCK_EI	3	8	14	22.8
ROSETTADOCK	0	0	1	61.5
ZRANK	4	6	8	40.9
ZRANK2	0	1	1	58.5
PYDOCK_TOT	8	14	18	17.3
SIPPER	0	0	0	94.1

S6 Comparison of per-residue and per-atom models

We compared the per-residue and per-atom representations used in ImaPEp. We started by analyzing the distance to the PCA plane, used as the fourth feature, and compared it in both representations. A distance value is associated with each residue in the per-residue model, and with each atom in the per-atom model. Thus, the distance of a given residue is a single value in the per-residue model; in the per-atom model, it is a set of N values (with N the number of heavy side chain atoms), where some atoms might have a normalized distance value very close to 1 and others, very close to 0. In such case, atoms with high distance values have high feature values (and thus appear brighter in the images) and those with close-to-zero distance values are nearly invisible.

The distance oversensitivity of the ImaPEp-atom model might be one of the reasons of its slightly lower average performance. An example in which this oversensitivity contributes to the correct classification of the entry is shown in Figure S1, where the paratope and epitope come from a complex between the H1N1 influenza virus neuraminidase of the 2009 pandemic and a neutralizing Ab (PDB ID: 4QNP). As seen in the box at the bottom left of the epitope images (b) and (e), the color of some atoms (in (e)) is brighter than the color of the residue (in (b)). This gives rise in the backward feature map to a darker red region in the per-atom model (f) compared to the same region in the per-residue model (c). In general, the problem is that atoms that are very close to the binding interface have a weight that is likely to be too strong and, on the average yield incorrect classifications.

In addition, the fine-granularity of the per-atom representation can also increase the probability of shape incompatibility of the represented interfaces in the images. As shown in Figure S1, the regions outlined by the top boxes in the images contain atoms in the epitope image (e) but not in the paratope image (d) in the per-atom model, whereas they contain residues in both the paratope image (a) and epitope image (b) in the per-residue model. This yields a negative (blue) contribution to the binding score of ImaPEp-atom (f), and a slightly positive (red) contribution in ImaPEp-resi (c). This contributes to the correct classification of the entry by the per-residue method and to its misclassification by the per-atom method.

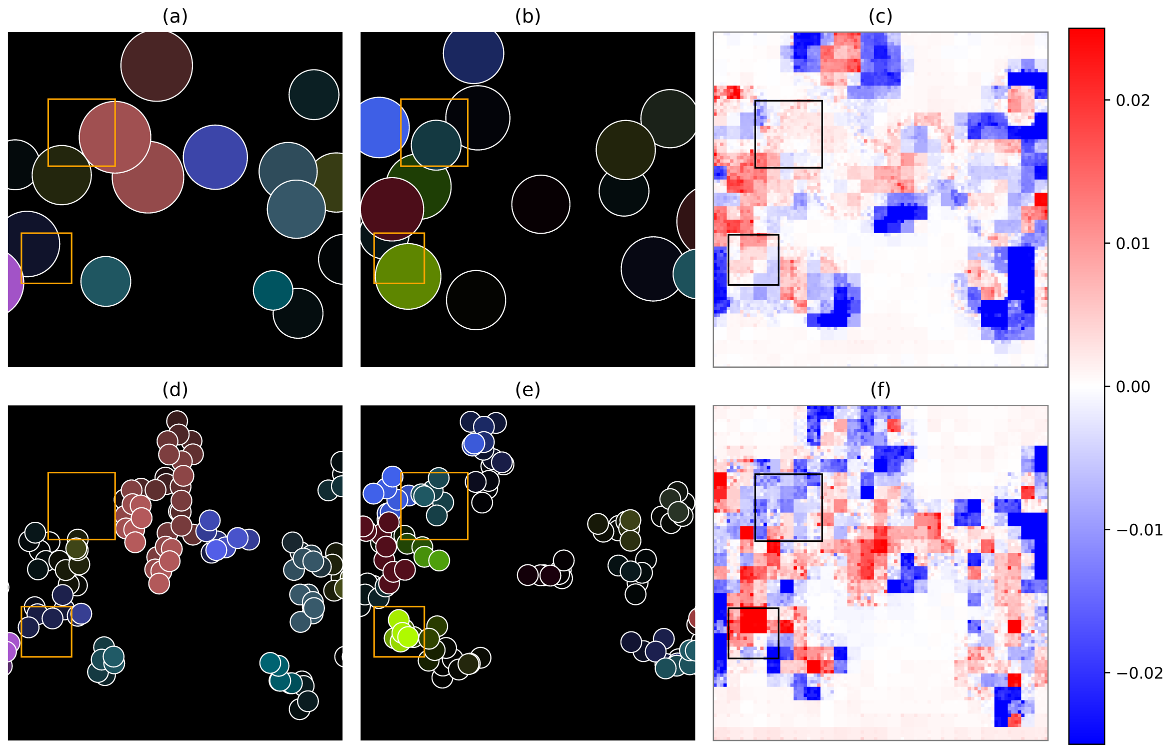

Figure S1

S7 3D distances in the 2D representation

It is widely accepted that protein-protein binding affinity is correlated with non-covalent interactions occurring at the interfaces, which are defined by shape complementarity and residue types. The goal of this analysis is to study whether our way of representing the Ab-Ag interfaces retains sufficient information about these interactions. In principle, the circles representing interacting residues with opposite charges in the epitope and paratope should have similar location in their respective images or a good alignment and the "distance" in the images should be related to the real distance between the two residues.

To analyze this, we selected four native Ab-Ag complexes (PDB IDs: 3MXW, 6ORO, 5ESV and 5JZ7) and identified the ionic interactions across the interface. For each interaction, we compared the sum of the distances of each of the two interacting residues to the PCA plane computed by ImaPEp with the real distances between the side chain geometric centers C_μ of these two residues. The result is shown in Table S5, and the paratope and epitope images of these complexes are shown in Figure S2.

We found that, overall, the residues that are in interaction are well aligned in the 2D representation of the paratope-epitope pairs, with sometimes a minor displacement of their geometrical position, as can be seen in Figure S2.a-d in which the residues involved in the ionic interactions listed in Table S5 are represented.

However, the distances computed by ImaPEp and used as features do not correspond exactly to the real distance observed in the 3D complex structure. The true distance between the interacting residues anticorrelates only weakly with the summed ImaPEp distance (Table S5). The reason for this lies in the 3D to 2D dimensional reduction to two PCA planes that, moreover, do not coincide.

Table S5: Analysis of the ionic interactions between paratope and epitope of Ab-Ag complexes with PDB IDs 3MXW, 6ORO, 5ESV and 5JZ7. The real distance is between the nitrogen carrying the positive charge and the oxygen carrying the negative charge, the C_μ distance is between side chain centroids, and the ImaPEp distance is defined in Section S2. Note that more positive ImaPEp distances correspond to atoms that are closer, whereas more negative ImaPEp distances correspond to atoms that are more distant.

PDB ID	Residue 1	Residue 2	Real distance (Å)	C_μ distance (Å)	ImaPEp distance (Å)
3MXW	Asp-A147	Arg-H098	2.6	5.47	-0.14
3MXW	Lys-A087	Glu-H032	3.0	5.89	1.05
3MXW	Arg-A153	Glu-H032	5.5	7.07	1.47
3MXW	Lys-A087	Glu-H097	2.7	5.04	5.18
3MXW	Arg-A153	Glu-H097	3.7	4.50	5.60
6ORO	Lys-G171	Asp-L050	2.8	5.34	-0.75
6ORO	Lys-G171	Asp-L053	5.9	5.53	0.22
5ESV	Lys-G171	Glu-H030	3.7	5.49	3.45
5JZ7	Arg-F059	Asp-I050	5.0	4.02	4.28

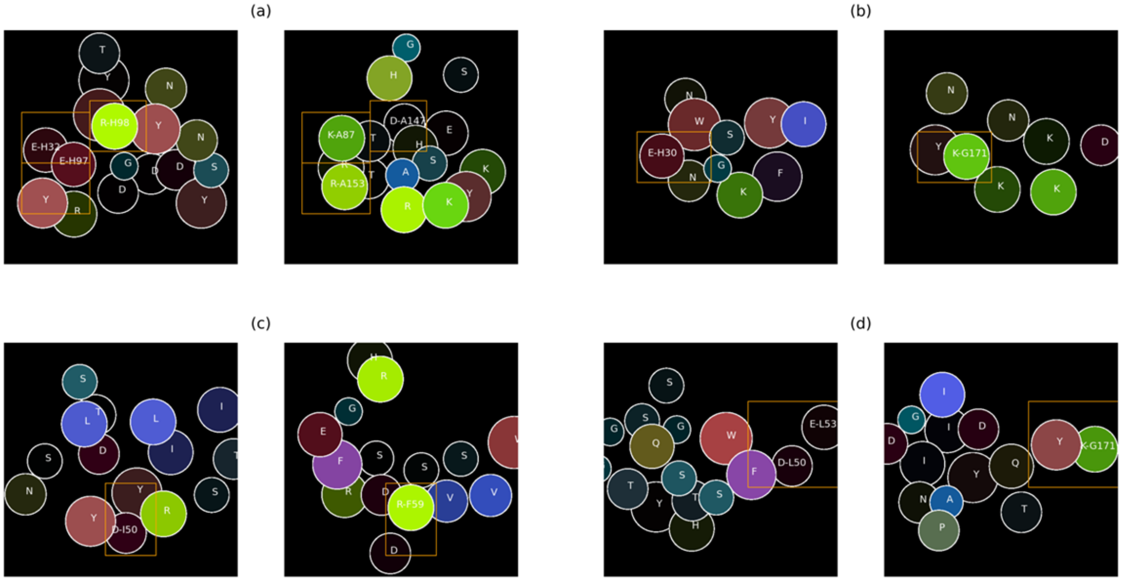

Figure S2: Images of the paratope-epitope interfaces of 3MXW (a), 5ESV (b), 5JZ7 (c) and 6ORO (d). For each image pair, the paratope image is on the left and the epitope image is on the right. The residues making a ionic Ag-Ab interaction are labelled using their one-letter code followed by their chain name and residue number, and outlined with orange rectangles. Other residues are only labelled with their amino acid code.

References

[1] W. Li and A. Godzik. Cd-hit: a fast program for clustering and comparing large sets of protein or nucleotide sequences. *Bioinformatics*, 22(13):1658–1659, July 2006.

[2] Limin Fu, Beifang Niu, Zhengwei Zhu, Sitao Wu, and Weizhong Li. CD-HIT: accelerated for clustering the next-generation sequencing data. *Bioinformatics*, 28(23):3150–3152, December 2012.

[3] Michael Levitt. A simplified representation of protein conformations for rapid simulation of protein folding. *Journal of molecular biology*, 104(1):59–107, 1976.

[4] Donard S Dwyer. Electronic properties of amino acid side chains: quantum mechanics calculation of substituent effects. *BMC Chemical Biology*, 5(1):1–11, 2005.

[5] Muhammad Idrees, Afzal R. Mohammad, Nazira Karodia, and Ayesha Rahman. Multi-modal role of amino acids in microbial control and drug development. *Antibiotics*, 9(6), 2020.

[6] Jack Kyte and Russell F Doolittle. A simple method for displaying the hydropathic character of a protein. *Journal of molecular biology*, 157(1):105–132, 1982.

[7] Pedro B. P. S. Reis, German P. Barletta, Luca Gagliardi, Sara Fortuna, Miguel A. Soler, and Walter Rocchia. Antibody-antigen binding interface analysis in the big data era. *Frontiers in Molecular Biosciences*, 9, 2022.

[8] Jean-Pierre A Kocher, Marianne J Rooman, and Shoshana J Wodak. Factors influencing the ability of knowledge-based potentials to identify native sequence-structure matches. *Journal of molecular biology*, 235(5):1598–1613, 1994.

[9] Iain H Moal, Brian Jiménez-García, and Juan Fernández-Recio. Ccharppi web server: computational characterization of protein–protein interactions from structure. *Bioinformatics*, 31(1):123–125, 2015.

[10] Keeley W Collins, Matthew M Copeland, Ian Kotthoff, Amar Singh, Petras J Kundrotas, and Ilya A Vakser. Dockground resource for protein recognition studies. *Protein Science*, 31(12):e4481, 2022.
